# Flight or protection: the genes *Ultrabithorax* and *apterous* in the determination of membranous and sclerotized wings in insects

**DOI:** 10.1098/rspb.2022.0967

**Published:** 2022-08-31

**Authors:** Moysés Elias-Neto, Niuska Alvarez, Alba Ventos-Alfonso, Xavier Belles

**Affiliations:** Institute of Evolutionary Biology, CSIC-Universitat Pompeu Fabra, Barcelona, Spain

**Keywords:** insect wings, *Ultrabithorax*, *Apterous*, *Blattella*, *tribolium*, wing specialization

## Abstract

Present-day pterygote insects have two pairs of wings, one in the mesothorax (T2), the other in the metathorax (T3), and both have diverged in structure and function in different groups. Studies in endopterygote and paraneopteran species have shown that the gene *Ultrabithorax* (*Ubx*) specifies the identity and wing structure in T3, whereas the gene *apterous* (*ap*) significantly contributes to forming modified T2 wings. We wondered whether these *Ubx* and *ap* mechanisms operate in the lineage of polyneopterans. To explore this possibility, we used the cockroach *Blattella germanica* (Polyneoptera and Blattodea), in which the T2 wings are sclerotized (tegmina), whereas those of the T3 are membranous. We found that *Ubx* determines the structure of T3 and the membranous wing, while *ap* significantly contributes to form the sclerotized T2 tegmina. These results along with the studies carried out on the beetle *Tribolium castaneum* by Tomoyasu and collaborators suggest that *ap* plays an important role in the sclerotization and melanization of the T2 wings in neopteran groups that have sclerotized forewings. In turn, the sclerotizing properties of *ap* demonstrated in beetles and cockroaches suggest that the origin of this function goes back to the emergence of Neoptera, in the mid Devonian.

## 1. Introduction

When Charles Darwin referred to the ‘endless forms most beautiful and most wonderful’, in the closing statement of his masterpiece ‘On the Origin of Species' [1], he was possibly thinking, above all, of insects, of which he was especially fond. The million plus species of insects described involve an extraordinary diversity of shapes, to a large extent owing to wing modifications. These particularly affect the forewings, which have adopted a spectacular variety of shapes and colours, often with protective functions. The present work deals with the mechanisms that configure the membranous or sclerotized structure of the insect wings.

Leaving secondary losses aside, pterygote insects have one pair of wings on the mesothorax (T2) and another on the metathorax (T3), which can be very different from each other. The first research into the mechanism that determines the shapes of the T2 and T3 wings was carried out on the fly *Drosophila melanogaster*, in which, as in most dipterans, the T2 wing is membranous and used for flight, while the T3 wing is a much smaller structure called a haltere, used to balance during flight. The spectacular ‘four-winged’ fly mutant phenotype obtained by Lewis [2] paved the way for further research. In this phenotype, the T3 of *D. melanogaster* is transformed into a practically complete copy of the T2, including the membranous wings. This dramatic transformation was induced by the loss-of-function mutations in the homeotic gene *Ultrabithorax* (*Ubx*), which specifies the identity of T3. Further studies revealed that the mechanisms underlying the action of *Ubx* in the T3 of *D. melanogaster* entail the repression of several genes involved in the formation of flying wings, and the stimulation of others contributing to the formation of halteres [3–7].

Subsequent RNA interference (RNAi) experiments depleting Ubx in the beetle *Tribolium castaneum* produced surprising results [8]. Considering antero-posterior prevalence as equivalent to phylogenetic sequence, what was generally expected was that the modified wing, the T2 elytra, would transform into a membranous wing under the systemic absence of Ubx. However, the opposite happened: the membranous T3 wings became sclerotized and melanized elytra. Thus, in beetles, as in flies, the segment (and its wings) specified by *Ubx* was T3. *Ubx* is expressed in T3 and also contributes to the differentiation of T3 wings in Lepidoptera, determining specific hindwing features, particularly eyespots, as reported in the butterflies *Precis coenia* [9] and *Bicyclus anynana* [10] (see also [11]). More recently, the T3-specifying effects of Ubx have been reported in the bug *Oncopeltus fasciatus* [12] and the planthopper *Nilaparvata lugens* [13]. In these hemipterans, RNAi depletion of Ubx resulted in T3 transforming into T2, so that the T3 membranous wings acquired the structure and colours of T2 hemelytra (*O. fasciatus*) or T2 diffusely thickened wings (*N. lugens*). The global effect of *Ubx* on an entire segment is associated with the chromatin modulator property demonstrated for this gene in *D. melanogaster* [14]. Through this property, Ubx increases and decreases chromatin accessibility, in line with its effect as an activator of haltere genes, and repressor of flying wing genes, as previously reported.

In terms of T2-modified wings, Tomoyasu *et al*. [15] working with the beetle *T. castaneum*, found that the elytra result from the activity of the basic set of wing genes plus those of other genes, including cuticular ones. Among all the genes, the important contribution of *apterous* (*ap*) stands out, since it is a key regulator of elytra sclerotization and melanization. The same authors showed that *T. castaneum* has two *ap* paralogues, *ap-A* and *ap-B*, originated from an ancestral gene duplication. Thus, insect species, in general, possess two *ap* paralogues, except for the genus *Drosophila* and possibly other brachycerans, in which *ap-B* was secondarily lost [15]. In the planthopper *N. lugens*, which has macropterous and brachypterous morphotypes, *ap-A* is significantly more expressed in macropterous nymphs. Moreover, the depletion of Ap-A in macropterous nymphs reduces the expression of the wing-related genes *Delta* (*Dl*), *spalt* (*sal*), *Serrate* (*Ser*), *vestigial* (*vg*) and *wingless* (*wg*), and results in adults with smaller wings [16]. More recently, Prakash & Monteiro [17], after mutating *ap-A* and *ap-B* in the butterfly *Bi. anynana*, showed that *ap-A*, which is expressed dorsally, acts both as a repressor and modifier of ventral wing colour patterns, as well as promoting dorsal sexual ornaments in males. The authors propose that the surface diversification of wing patterns in butterflies operated through the co-option of *ap-A* (or its downstream effectors) into different gene networks associated with the differentiation of discrete wing traits [17].

The vast majority of flying insects (pterygotes) belong to the neopteran division, which emerged some 380 Ma. The most typical feature of neopterans is that they can flex their wings over the dorsal body side. In turn, the neopterans are divided into three main lineages that emerged almost simultaneously some 25 Myr later: polyneopterans (which includes grasshoppers, crickets, cockroaches, termites, stick-insects and stoneflies, for example), paraneopterans (true bugs, planthoppers, whiteflies, aphids, cicadas, lice and thrips) and endopterygotes (beetles, butterflies, moths, ants, bees, flies and mosquitoes among other groups) [18]. The data compiled above shows the key role of *Ubx* in the specification of T3 and its wings, and the important role of *ap* in the modification of T2 wings. However, those studies have been carried out on flies, beetles and butterflies, among the endopterygotes, as well as true bugs and planthoppers, representing the paraneopterans. Therefore, the data do not cover any species from the polyneopteran lineage, with some 125 000 species described [19]. The question that arises is, are the *Ubx* and *ap* mechanisms related to T3 and T2 wing formation also present in the important lineage of polyneopterans? This work aims to answer that question using the cockroach *Blattella germanica* (Polyneoptera and Blattodea) as a model.

## 2. Methods

### (a) Insects

*Blattella germanica* specimens used in the experiments were obtained from a colony reared in the dark at 30 ± 1°C and 60–70% relative humidity. They were carbon dioxide-anaesthetized prior to dissections and tissue sampling.

### (b) RNA extraction and retrotranscription to complementary DNA

All RNA extractions were carried out with the Gen Elute Mammalian Total RNA kit (Sigma-Aldrich, Madrid, Spain). An amount of 400 ng from each RNA extraction was treated with DNase (Promega, Madison, WI, USA) and reverse transcribed with Superscript II reverse transcriptase (Invitrogen, Carlsbad CA, USA) and random hexamers (Promega). RNA quantity and quality were estimated by spectrophotometric absorption at 260 nm in a Nanodrop Spectrophotometer ND-1000^®^ (NanoDrop Technologies, Wilmington, DE, USA).

### (c) Determination of messenger RNA levels with quantitative real-time polymerase chain reaction

Quantitative real-time polymerase chain reactions (PCRs) were carried out in triplicate in an iQ5 Real-Time PCR Detection System (Bio-Rad Laboratories, Madrid, Spain), using SYBR^®^ Green (Power SYBR^®^ Green PCR Master Mix; Applied Biosystems, Madrid, Spain). A control without a template was included in all batches. The primers used for each transcript measured are described in the electronic supplementary material, table S1. The efficiency of each primer set was first validated by constructing a standard curve through four serial dilutions. Messenger RNA (mRNA) levels were calculated relative to actin-5c and EIF4A expression using the Bio-Rad iQ5 Standard Edition Optical System Software (v. 2.0). The primers used to quantify actin-5c and EIF4A are indicated in the electronic supplementary material, table S1. We followed a method based in threshold-cycle (Ct) according to the Pfaffl mathematical model [20], simplifying to 2^ΔΔCt^ because the calculated efficiency values for studied genes and actin-5c/EIF4A amplicons were always within the range of 95 to 100%; therefore, no correction for efficiency was used in further calculations. Results are given as copies of mRNA per 1000 copies of actin-5c/EIF4A mRNA. Statistical differences between groups were tested by the REST 2008 program (Relative Expression Software Tool v. 2.0.7; Corbett Research) [20].

### (d) RNA interference

Detailed procedures for double stranded RNA (dsRNA) preparation and RNAi experiments were as described previously [21,22]. RNAi experiments were used to target *Ubx*, *ap-A* and *ap-B* transcripts. The primers used to prepare the corresponding dsRNAs are detailed in the electronic supplementary material, table S1. In all cases, we used a 307 bp sequence from *Autographa californica* nucleopolyhydrosis virus as control dsRNA (dsMock). In the single interference targeting *Ubx*, *ap-A* or *ap-B*, a volume of 1 µl of dsRNA solution (3–5 µg µl^−1^) either of the targeted gene (dsUbx, dsAp-A or dsAp-B) or of control (dsMock) was injected into the abdomen of nymphs. Two successive treatments were made in the fifth and sixth (last) nymphal instar, freshly emerged in all cases. In the case of double interference targeting *ap-A* and *ap-B*, the treatments were a mixture of 0.5 µl of dsAp-A solution (6 µg µl^−1^) plus 0.5 µl of dsAp-B solution (6 µg µl^−1^), and, as control, 1 µl of dsMock solution (6 µg µl^−1^).

### (e) Wing morphological studies

Adult forewings (tegmina) and hindwings (membranous) were studied and photographed first in the intact animal, and then dissected out, mounted on a slide with Mowiol 4–88 (Sigma-Aldrich, Madrid, Spain). Examinations and photographs were made with a stereomicroscope Zeiss DiscoveryV8. Biometrical measurements of wing and leg size parameters were carried out with an ocular micrometer adapted to this stereomicroscope.

## 3. Results

### (a) *Ultrabithorax* specifies the hindwing features

**Figure 1.**
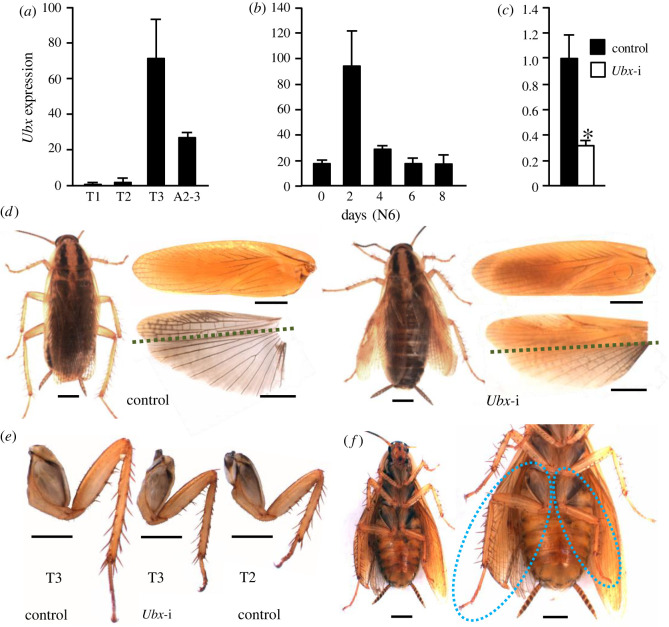
Effects of *Ultrabithorax* (*Ubx*) depletion on wing development in *Blattella germanica*. (*a*) *Ubx* expression in the prothorax (T1), mesothorax (T2), metathorax (T3) and the second and third abdominal segments (A2–3) of 2-day-old sixth nymphal instar (N6D2). (*b*) Expression pattern of *Ubx* in T3 during the sixth nymphal instar (N6). (*c*) *Ubx* expression in N6D2 in controls and dsUbx-treated (*Ubx*-i) insects. (*d*) General view of the adult female, and detail of the T2 tegmina (upper part) and T3 membranous wing (lower part) in control and Ubx-depleted insects (*Ubx*-i); the dashed line in the T3 wing of control and *Ubx*-i specimens roughly separates the remigium (above the line) and the anal (below) regions. (*e*) Adult T3 leg in *Ubx*-i insects compared with controls; a T2 hind leg from control insects is also shown for comparison. (*f*) *Ubx*-i adult female with asymmetric transformation of the T3; notice the difference in size of the right hind leg, typical of the T3 segment and the smaller left leg, such as that of the T2 segment; in the enlarged figure on the right, the two hind legs are highlighted with an oval of dots. In (*a*,*c*), expression is represented as mRNA copies per 1000 copies of actin-5c (mean ± s.e.m., *n* = 3–4); in (*c*), the asterisk indicates statistically significant differences with respect to controls (*p* < 0.05), according to REST [20]. In (*d*,*f*), scale bars: 2 mm. (Online version in colour.)

As previously observed [22], *Ubx* expression in *Bl. germanica* concentrates in T3, although significant expression can also be measured in the first abdominal segments (figure 1*a*). The expression pattern in T3 in the sixth (last) nymphal instar shows an acute peak on day 2, whereas on the other days of the instar, the expression is about five times lower (figure 1*b*).

To study the function of *Ubx*, we used nymphal RNAi. Female nymphs were treated with two doses of 5 µg of dsUbx, the first injected into the fifth nymphal instar just after emergence (N5D0) and the second into N6D0. Controls were treated equivalently with dsMock. The dsUbx treatment significantly reduced the *Ubx* transcript levels in T3, as measured in N6D2 (figure 1*c*). Both control (*n* = 28) and dsUbx-treated insects (*n* = 35) moulted to adults after N6D8. Controls moulted to normal adults, with well-patterned and correctly extended T2 and T3 wings. By contrast, the dsUbx-treated insects moulted to adults with wings that did not perfectly extend. Moreover, the T3 wings showed coloration and hardness alterations (figure 1*d*). Dissected and extended onto a slide, the shape and venation of the T2 tegmina of Ubx-depleted adults were similar to those of the controls. By contrast, the T3 wings were modified by the dsUbx treatment. The remigium area was sclerotized and yellowish, with a vein patterning reminiscent of tegmina, whereas the anal area was reduced, being more sclerotized and yellowish, especially in the region adjacent to the remigium, and with altered vein patterning, with a high number of atypical transversal veins (figure 1*d*; electronic supplementary material, table S2). Apparently, the dsUbx treatment affected the entire T3 segment, which became similar to the T2, as shown by the shape and size of the legs, which were similar to T2 legs (figure 1*e*). These observations suggest that the dsUbx treatment triggered a homeotic transformation of T3 into T2. Curiously, one of the 35 dsUbx-treated insects moulted to an adult with an asymmetrical thorax, the right half similar to a normal T3, and the left more similar to a T2 (figure 1*f*).

### (b) *Apterous* has two paralogues in *Blattella germanica* which are expressed at very different levels

Given that other insect species, such as *T. castaneum* [15], have two *ap* paralogues, we wanted to confirm whether this also occurs in *Bl. germanica*. Our search of the genome [23] and available transcriptomes [24] led to us identifying two *ap* paralogues in *Bl. germanica*, *ap-A* and *ap-B* (electronic supplementary material, figure S1).

**Figure 2.**
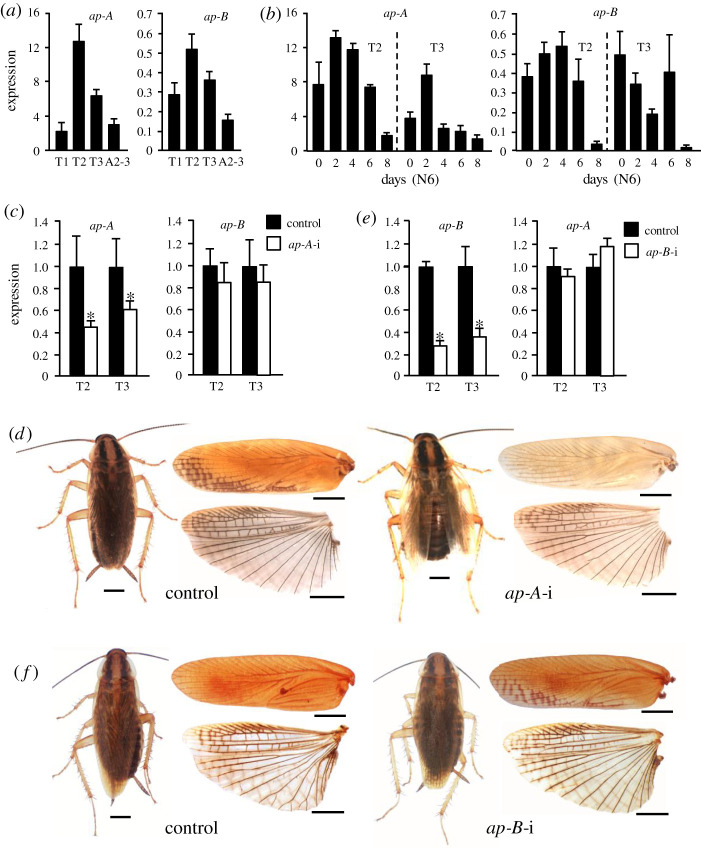
Effects of the depletion of *apterous-A* (*ap-A*) and *apterous-B* (*ap-B*) on wing development in *Blattella germanica*. (*a*) *ap-A* and *ap-B* expression in the prothorax (T1), mesothorax (T2), metathorax (T3), and the second and third abdominal segments (A2–3) of 2-day-old sixth nymphal instar (N6D2). (*b*) Expression pattern of *ap-A* and *ap-B* during the sixth nymphal instar. (*c*) *ap-A* and *ap-B* expression in N6D2 in controls and dsAp-A-treated (*ap-A*-i) insects. (*d*) General view of the adult female, and detail of the T2 tegmina (upper part) and T3 membranous wing (lower part) in control and *ap-A*-i insects. (*e*) *ap-B* and *ap-A* expression in N6D2 in controls and dsAp-B-treated (*ap-B*-i) insects. (*f*) General view of the adult female, and detail of the T2 tegmina (upper part) and T3 membranous wing (lower part) in control and *ap-B*-i insects; the tegmina from the *ap-B-i* insect shown in the panel is one of the 12 (out of 32) that had the posterior edge, at the distal end, slightly depigmented. In (*a*–*c*) and (*e*), expression is represented as mRNA copies per 1000 copies of actin-5c (mean ± s.e.m., *n* = 3–4); in (*c*) and (*e*), the asterisk indicates statistically significant differences with respect to controls (*p* < 0.05), according to REST [20]. In (*d*) and (*f*), scale bars: 2 mm. (Online version in colour.)

First, we studied the expression of *ap-A* and *ap-B* in the thoracic and first abdominal segments. The results showed that the highest level of expression occurs in T2, followed by T3 for both *ap-A* and *ap-B*, although the respective levels were more than one order of magnitude higher in the case of *ap-A* (figure 2*a*). The expression pattern in T2 and T3 during the sixth nymphal instar shows an increase to maximum values between days 2 and 4, and a progressive decrease until day 8. The expression profile of *ap-B* in T3 seems divergent, with two peaks, on day 0 and day 6, but the notable dispersion of values on those days suggests that the pattern might not be very different from that observed in T2. Again, these results confirm that *ap-A* is expressed at higher levels than *ap-B* (figure 2*b*).

### (c) *Apterous* contributes to specifying the mesothoracic tegmina

To investigate the role of *ap* in terms of wing formation, we also used nymphal RNAi. In a first set of experiments, we targeted *ap-A* using a specific region of the corresponding transcript sequence to design the dsAp-A. Female nymphs were treated with two doses of 3 µg of dsAp-A, the first injected into N5D0 and the second into N6D0. Controls were treated equivalently with dsMock. The dsAp-A treatment significantly reduced the *ap-A* transcript levels in T2 and T3, as measured in N6D2; conversely, *ap-B* transcript levels were not affected (figure 2*c*). Both control (*n* = 34) and dsAp-A-treated insects (*n* = 38) moulted to adults after N6D8, but whereas the controls moulted to normal adults, with well-patterned and correctly extended T2 and T3 wings, the dsAp-A-treated insects moulted to adults with alterations in the T2 tegmina, which were less pigmented, softer and less sclerotized than those of the controls. The T3 membranous wings were practically unaffected by the dsAp-A treatment, although a few specimens presented a somewhat browner remigium area than in the controls, and the entire wing was more fragile, cracking easily when handled during dissection. In terms of wing shape and venation pattern, no outstanding alteration was observed in relation to the controls, in either the T2 tegmina or the T3 membranous wings (figure 2*d*; electronic supplementary material, table S2).

Next, we targeted the *ap-B* transcripts using a dsAp-B designed in a specific region of the *ap-B* sequence. Female nymphs were treated in the same way as in the RNAi experiments targeting *ap-A*. The dsAp-B treatment significantly reduced the *ap-B* transcript levels in T2 and T3, as measured in N6D2; conversely, *ap-A* expression was not significantly affected (figure 2*e*). Both control (*n* = 27) and dsAp-B-treated insects (*n* = 32) moulted to adults after N6D8. The insects from both groups moulted to normal adults, with well-patterned and correctly extended T2 and T3 wings. Looking at the T2 and T3 wings in detail, practically no differences were discerned between those of the dsAp-B-treated insects and the controls. At most, some specimens (12 out of 32) showed the posterior edge, at the distal end, slightly depigmented (figure 2*f*; electronic supplementary material, table S2).

**Figure 3.**
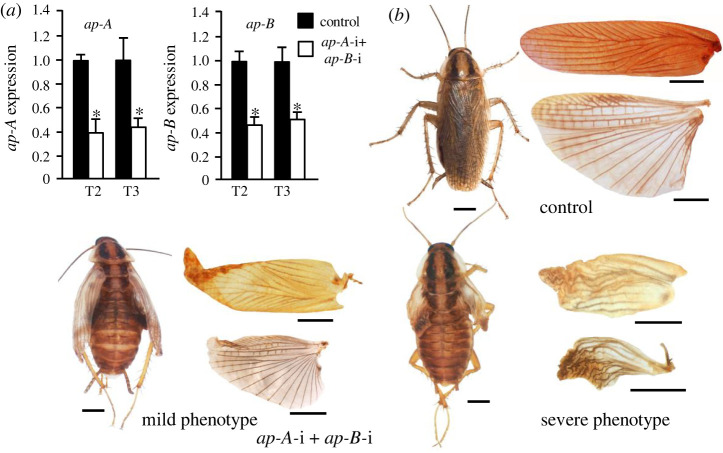
Effects of *apterous-A* and *apterous-B* joint depletion on wing development in *Blattella germanica*. (*a*) *ap-A* and *ap-B* expression in N6D2 in the mesothorax (T2) and metathorax (T3) of control and dsAp-A + dsAp-B-treated (*ap-A*-i + *apB*-i) insects. (*b*) General view of the adult female, and detail of the T2 tegmina (upper part) and T3 membranous wing (lower part) in control and *ap-A*-i + *apB*-i insects; in the latter, mild and severe phenotypes have been differentiated. In (*a*), expression is represented as mRNA copies per 1000 copies of actin-5c (mean ± s.e.m., *n* = 3–4); the asterisk indicates statistically significant differences with respect to controls (*p* < 0.05), according to REST [20]. In (*b*), scale bar: 2 mm. (Online version in colour.)

Finally, the transcripts of the two paralogues, *ap-A* and *ap-B*, were targeted at the same time. N5D0 female nymphs were treated with a mixture of dsAp-A and dsAp-B (3 µg each in a volume of 1 µl), and the treatment was repeated in N6D0. Controls were treated equivalently with 6 µg of dsMock (in a volume of 1 µl) in N5D0 and again in N6D0. Transcript changes were measured in N6D2, showing that the treatment efficiently reduced the levels of both transcripts, *ap-A* and *ap-B* (figure 3*a*). Both controls (*n* = 21) and insects treated with dsAp-A and dsAp-B (*n* = 28) moulted to adults after N6D8. As expected, the controls moulted to normal adults. By contrast, the dsAp-A + dsAp-B-treated insects moulted to adults with wing alterations (figure 3*b*). A total of 18 out of the 28 treated insects (64%) showed a mild phenotype, with the wings being similar to those obtained with *ap-A* depletion, i.e. the T2 tegmina were less pigmented, softer and less sclerotized than in the controls, and the T3 membranous wings were somewhat brownish in colour, mainly in the remigium area. By contrast, 10 out of the 28 treated insects (36%) showed a severe phenotype, with dramatically reduced T2 and T3 wings, which were wrinkled and brittle (figure 3*b*; electronic supplementary material, table S2).

### (d) *Ultrabithorax* and *apterous-A* transcript depletion have a differential effect on other wing-related genes

To shed light on the pathways of gene expression in the phenotypes observed when depleting the *Ubx* and *ap-A* transcripts, we studied the effect of such depletions on the expression of the following wing-related genes: *blistered* (*bs*), *cut* (*ct*), *Dl*, *decapentaplegic* (*dpp*), *Epidermal growth factor receptor* (*Egfr*), *engrailed* (*en*), *Notch* (*N*), *nubbin* (*nub*), *rhomboid* (*rho*), *spalt major* (*salm*), *scalloped* (*sd*), *Ser*, *vg* and *wg*. The expression levels of these genes in the three thoracic segments of *Bl. germanica* had been published previously [22]. We followed the same protocol of nymphal RNAi for *Ubx* and *ap-A* as described above, using the same doses of dsRNA and treatments in N5D0 and N6D0. We were interested in the role of *ap* on tegmina sclerotization, and the most informative treatment regarding this role was obtained by depleting *ap-A* (figure 2*d*). Therefore, we chose the dsAp-A treatment to study the effect on other genes. By contrast, the depletion of *ap-B* did not produce a significant morphological phenotype, and joint depletion of *ap-A* and *ap-B* affected entire wing development, well beyond the effect on tegmina sclerotization (figure 3*b*). Therefore, we did not use these treatments in this part of the work.

**Figure 4.**
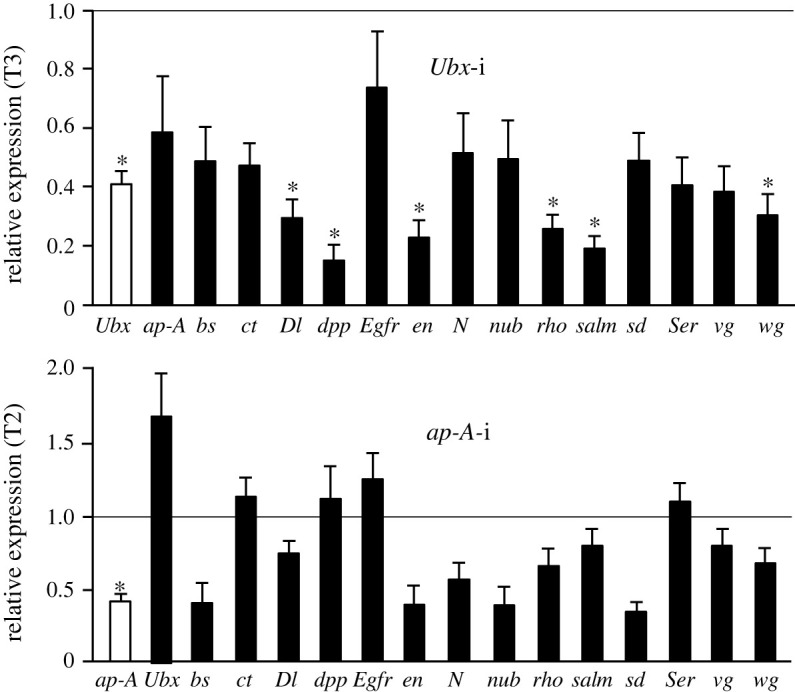
Effects of dsUbx and dsAp-A treatment depletion on the expression of other wing-related genes in *Blattella germanica*. The effects of *ap-A* and *Ubx* transcript depletion (*Ubx*-i and *ap-A*-i, respectively) on the expression of *blistered* (*bs*), *cut* (*ct*), *delta* (*Dl*), *decapentaplegic* (*dpp*), *epidermal growth factor receptor* (*Egfr*), *engrailed* (*en*), *Notch* (*N*), *nubbin* (*nub*), *rhomboid* (*rho*), *spalt major* (*salm*), *scalloped* (*sd*), *Serrate* (*Ser*), *vestigial* (*vg*) and *wingless* (*wg*) are shown. Data represent normalized values against control (dsMock-treated) (reference value = 1) and is expressed as mRNA copies per 1000 copies of EIF4A (mean ± s.e.m., *n* = 3–4); the asterisk indicates statistically significant differences with respect to controls (*p* < 0.05) according to REST [20]. Reference expression values of these genes in the three thoracic segments of *Bl. germanica* have been reported previously [22]. (Online version in colour.)

The results (figure 4) indicate that *Ubx* depletion leads to a downregulation of the expression of all the genes measured in T3. The differences with respect to the controls were statistically significant in the case of *Dl*, *dpp*, *en*, *rho*, *salm* and *wg*. Interestingly, the levels of these last four started to attain values similar to those they have in the T2 segment [22]. According to the same study [22], the expression of *bs* and *vg* is lower in T2 than in T3, and, although not significantly different from the controls, the mRNA levels of these two genes tend to be lower in Ubx-depleted insects, reaching levels that are similar to those of T2. The depletion of Ap-A caused no statistically significant changes in the expression of the genes measured in T2. Most genes tended to decrease their expression levels, especially *sd*, *en*, *nub* and *bs*, with a *p*-value of between 0.1 and 0.05. However, some of them became upregulated, notably *Ubx*, which almost doubled its expression values, bringing them closer to those of T3 [22], a segment characterized by high expression of *Ubx* and membranous wings.

## 4. Discussion

### (a) *Ultrabithorax* and the membranous hindwings

In *Bl. germanica*, *Ubx* is predominantly expressed in T3, showing a transcript level peak on day 2 of the last nymphal instar. Functionally, Ubx determines the identity of T3, including the membranous hindwings. These results are reminiscent of those originally reported in *D. melanogaster* by Lewis [2], where Ubx specifies the identity of T3, determining the formation of the modified halteres [3–7]. However, our results are more similar to those described in the beetle *T. castaneum* [8] and in the hemipterans *O. fasciatus* [12] and *N. lugens* [13], species in which the modified wings are the T2, which become elytra (*T. castaneum*), hemelytra (*O. fasciatus*) or diffusely thickened wings (*N. lugens*). In those species (as in *Bl. germanica*, in which the T2 wing have become tegmina), Ubx determines the formation of membranous wings in T3 (also see [25] for a review).

In general, the suppression or depletion of Ubx results in a homeotic transformation of T3 into T2, which is observed not only in the wings but also in the legs, which adopt the shape and size of T2 legs. We also observed this in *Bl. germanica*, in which there are no relevant morphological differences between T2 and T3 legs, and only the difference in size is apparent, the hind legs being longer than the mid legs. The transformation of T3 legs into T2 legs after Ubx depletion has been observed in other species in which T3 legs are modified, such as the honeybee *Apis mellifera* [26,27], the cricket *Acheta domestica* [28] and the hemipterans *O. fasciatus* [28] and *N. lugens* [13]. Finally, in *O. fasciatus* [29] and the lepidopteran *Bombyx mori* [30], RNAi of *Ubx* resulted in the first abdominal segment taking on the identity of T3. However, we observed no changes in the abdominal segments after Ubx depletion in *Bl. germanica*.

### (b) *Apterous* and the sclerotized tegmina

We found that like almost all insects (except in *Drosophila* species) [15], *Bl. germanica* has two *ap* paralogues: *ap-A* and *ap-B*. However, the expression levels of *ap-A* are more than an order of magnitude higher than those of *ap-B*. Moreover, *ap-A* and *ap-B* expression is highest in T2 and slightly lower in T3.

In *Bl. germanica*, *ap-A* depletion produced a loss of sclerotization and colour in the T2 tegmina, while that of *ap-B* had no significant effects. These results, and the lower expression levels of *ap-B* compared to *ap-A*, suggest that the functions of the two genes are redundant. However, the joint depletion of *ap-A* and *ap-B* produced a more severe phenotype than with just *ap-A* depletion, resulting in a dramatic reduction of both the T2 and the T3 wings in a significant number of treated insects. Taken together, the results suggest that *ap* contributes to the formation and stretching of T2 and T3 wings and, additionally, to the sclerotization and melanization of the T2 tegmina. Our results are similar to those obtained for *T. castaneum*, where the mildest phenotype of *ap* depletion (obtained with late RNAi) is a defect in T2 elytra sclerotization, while the most severe (obtained with early RNAi, especially when depleting both *ap-A* and *ap-B* at the same time) is the virtual absence of elytra in T2 and membranous wings in T3 [15].

### (c) *Ultrabithorax, apterous* and other wing-related genes

In T3, Ubx depletion causes a decrease in the expression of all the wing-related genes studied, in particular *Dl*, *dpp*, *en*, *rho*, *salm* and *wg*, the drop in which compared to controls is statistically significant. The stimulatory effect of Ubx on these wing-related genes in *Bl. germanica* contrasts with the repressive effect of Ubx on the expression of a series of wing-related genes in the T3 of *D. melanogaster* [3–7], which results in the formation of the haltere. By contrast, the activating effect of Ubx on T3 wing-related genes that we observed in *Bl. germanica* is reminiscent of the situation in *T. castaneum*, where the orthologues of the wing-related genes that Ubx represses in *D. melanogaster* T3, like, for example*, sal*, are activated by Ubx in *T. castaneum* [8].

In *Bl. germanica* T2, Ap-A depletion results in an increase or decrease in the expression of wing-related genes, according to each gene, although differences with respect to the controls are not statistically significant in any case. Most genes tend to be downregulated, such as *bs*, *en*, *N*, *nub*, *rho*, *sd*, and *wg*, which suggests that Ap stimulates the expression of wing-related genes in the tegmina, and that these are still necessary in this modified wing, as happens with elytra in beetles [15]. In some genes, the expression tends to be upregulated by the Ap-A-depletion. Among these, the almost twofold increase in *Ubx* expression is interesting as it may explain, at least in part, the ‘membranization’ of the tegmina of Ap-A-depleted insects. Thus, Ap proteins appear to repress *Ubx* expression in T2, facilitating the formation of tegmina instead of membranous wings.

The more dramatic effect of *Ubx*, compared to *ap-A*, on the expression of other genes can be explained by the modulatory role of chromatin that has been characterized for this gene in *D. melanogaster* [14]. Thus, Ubx can increase and decrease chromatin accessibility, which fits with its dual role as both an activator and repressor of transcription that specifies the identity of T3 and the formation of halteres in the fly. This chromatin-modulating mechanism is what underlies the concept of ‘micromanager’ for haltere formation, proposed by Akam [31], and commented further by Tomoyasu [25].

### (d) *Ultrabithorax*, *apterous* and the evolution of wing diversity in insects

Until now, the role of the Hox gene *Ubx* as a specifier of the T3 segment and the corresponding wing, and that of the selector gene *ap* in contributing to determining T2 features and eventual modifications had been functionally studied in flies (Diptera), beetles (Coleoptera) and butterflies (Lepidoptera), among the Endopterygota, and in true bugs and planthoppers (Hemiptera), among the Paraneoptera. Thus, the mechanisms associated with *Ubx* and *ap* in wing development were known to operate in the Eumetabola, a lineage comprising the Endopterygota and Paraneoptera. Our finding that the modulating action of *Ubx* and *ap* on T3 and T2 wings also operates in cockroaches (Blattodea), extends the wing-related effects of these two genes to Polyneoptera and, therefore, to all Neoptera lineages.

The evolutionary history of insects indicates that wings appeared in the early Devonian, some 410 Ma. According to the fossil record, the first winged insects would have had approximately equal T2 and T3 membranous wings, perpendicular to the body axis, such as those of present-day dragonflies and mayflies (Palaeoptera), whose shape and simple articulation allow them to flap up and down for flying. A subsequent innovation was wing flexion, which appeared in the neopterans, some 30 Ma later. This involves the rotation of wings around the articulation tergum sclerite, permitting the wing to flex over the dorsal side of the body [18,32,33].

The role of *Ubx* in specifying the T3 segment seems to predate the origin of the Neoptera, even that of the Pterygota, since in the wingless *Thermobia domestica* (Zygentoma), the Ubx protein is concentrated in T3, whereas only very low levels are observed in T2 [34]. This suggests that the situation could be similar in the Palaeoptera, although the two pairs of wings are membranous and similar to one another in this lineage. It is from the neopterans onwards that a notable diversification of the T2 wings began, often involving sclerotization processes, which gave the forewings a generally protective function. Clear examples of this are the elytra of beetles, the hemelytra of true bugs and tegmina in cockroaches. The results obtained in the functional experiments carried out on the beetle *T. castaneum* [15] and the cockroach *Bl. germanica* (this study) suggest that *ap* has played an important role in the sclerotization of the T2 wings in neopteran groups with sclerotized forewings. These results also suggest that this ‘sclerotizing’ action on T2 wings dates back to the origin of the Neoptera. Of course, the contributions of other co-opted genes would be added to the common action of *ap*. These co-opted genes would be specific to each group with sclerotized forewings and would confer the corresponding particular features. The thoroughly studied case of the different cuticular protein gene expressed in the elytra of *T. castaneum* [15] is a good example of repeated co-option in a single species. The action of *ap* and the genes specifically co-opted in each insect group would result in the explosion of forewing forms and colours that we witness today in different neopteran lineages.

## Data Availability

The data are provided in the electronic supplementary material [35].
